# Autism Spectrum Disorder in an Unselected Cohort of Children with Neurofibromatosis Type 1 (NF1)

**DOI:** 10.1007/s10803-018-3478-0

**Published:** 2018-02-08

**Authors:** S. Eijk, S. E. Mous, G. C. Dieleman, B. Dierckx, A. B. Rietman, P. F. A. de Nijs, L. W. ten Hoopen, R. van Minkelen, Y. Elgersma, C. E. Catsman-Berrevoets, R. Oostenbrink, J. S. Legerstee

**Affiliations:** 1 Department of Child and Adolescent Psychiatry/Psychology, Erasmus Medical Center-Sophia Children’s Hospital, P.O. Box 2060, 3000 CB Rotterdam, The Netherlands; 2 ENCORE Expertise Center for Neurodevelopmental Disorders, Erasmus Medical Center Sophia Children’s Hospital, P.O. Box 2060, 3000 CB Rotterdam, The Netherlands; 3 Department of Clinical Genetics, Erasmus Medical Center, P.O. Box 2040, 3000 CA Rotterdam, The Netherlands; 4 Department of Neuroscience, Erasmus Medical Centre Rotterdam, 3015 CN Rotterdam, The Netherlands; 5 Department of Pediatric Neurology, Erasmus Medical Center-Sophia Children’s Hospital, P.O. Box 2060, 3000 CB Rotterdam, The Netherlands; 6 Department of General Paediatrics, Erasmus Medical Center-Sophia Children’s Hospital, P.O. Box 2060, 3000 CB Rotterdam, The Netherlands; 7 Department of Child and Adolescent Psychiatry/Psychology, Erasmus Medical Center-Sophia Children’s Hospital, Room Sp-2509, P.O. Box 2060, 3000 CB Rotterdam, The Netherlands

**Keywords:** Neurofibromatosis type 1, Autistic traits, Autism spectrum disorder, Prevalence, Autism diagnostic observation schedule, Social responsiveness scale

## Abstract

In a non-selected sample of children with Neurofibromatosis type 1 (NF1) the prevalence rate of autism spectrum disorder (ASD) and predictive value of an observational (ADOS)—and questionnaire-based screening instrument were assessed. Complete data was available for 128 children. The prevalence rate for clinical ASD was 10.9%, which is clearly higher than in the general population. This prevalence rate is presumably more accurate than in previous studies that examined children with NF1 with an ASD presumption or solely based on screening instruments. The combined observational- and screening based classifications demonstrated the highest positive predictive value for DSM-IV diagnosis, highlighting the importance of using both instruments in children with NF1.

## Introduction

Neurofibromatosis type 1 (NF1) is an autosomal dominant disorder affecting 1 in 2500–3000 individuals (Williams et al. 2009). The disorder is inherited in half of the cases, and in the other half the mutation is de novo (Messiaen et al. 2000). The NF1 gene encodes for the protein neurofibromin, which activates the protein RasGTPase (Rauen 2013). RasGTPase functions as a negative regulator of Ras, a protein involved in the regulation of the cell cycle, growth and differentiation. As a result of mutations in NF1, a decrease in neurofibromin activity causes increased cell growth. Affected individuals are recognized by the representation of at least two distinctive physical features, including café-au-lait spots, intertriginous freckling, Lisch nodules, neurofibromas, optic pathway gliomas or distinctive bone-forming lesions (Williams et al. 2009).

Children with NF1 often experience cognitive and behavioral problems (Hachon et al. 2011; Lehtonen et al. 2013). Generally, intelligence scores of affected children are significantly lower compared to the general population, and learning problems and attention-deficit-hyperactivity disorder (ADHD) are common. However, the problems are highly variable across the NF1 population (Lehtonen et al. 2013). Besides these common cognitive and behavioral characteristics, social difficulties have been reported in children with NF1. Children with NF1 often have poorer social skills, tend to be socially isolated and rejected by peers, and experience problems in social information processing (Barton and North 2004; Huijbregts et al. 2010; Noll et al. 2007). Similar to the general population, social problems in children with NF1 are more prevalent in boys (Garg et al. 2016) and in children with low intellectual functioning (Huijbregts and De Sonneville 2011).

Recently, studies have focused on the prevalence and profile of autism spectrum disorders (ASD) in children with NF1. Compared to global ASD prevalence estimates of 0.8% in the general population (Baxter et al. 2015) screening-based prevalence rates of clinical ASD symptoms of 13–29% have been found in children with NF1. On top of this, an additional percentage of 27–31% of children were found to show subclinical symptoms, leading to total estimated screening-based prevalence rates of ASD symptoms ranging between 30–56% (Adviento et al. 2014; Constantino et al. 2015; Garg et al. 2013; Van Eeghen et al. 2013; Walsh et al. 2013). In a recent, internationally compiled sample of children with NF1 (*N* = 531), a screening-based prevalence rate of clinical ASD of 13% and an additional prevalence rate of 26% of subclinical symptoms was reported (Morris et al. 2016), resulting in a total prevalence rate of ASD traits of 39%. It should be noted, though, that these estimates are based on screening instruments. Because of their measurement purposes, these instruments are highly sensitive and thus may result in a biased (slightly overestimated) ASD prevalence rate.

The possible overestimation of the screening-based prevalence rates highlights the importance of studies assessing clinical ASD prevalence rates. Only a few studies have examined clinically assessed ASD prevalence rates in children with NF1. In the studies by Garg et al. (2013) and Plasschaert et al. (2015), children with a presumption of ASD (preselected based on elevated scores on a screening instrument) were assessed with clinical diagnostic instruments. In these subsamples of children with NF1, ASD prevalence estimates of 25% (Garg et al. 2013) and 26% (Plasschaert et al. 2015) were reported. Because these prevalence rates were based on samples of children with an initial suspicion of autism spectrum problems, these prevalence rate are probably not representative for the general pediatric NF1 population as a whole. To our knowledge, there are no reports available in the literature in which an unselected sample of children with NF1 has been clinically assessed for ASD.

The primary aim of this study was to examine the prevalence of clinically assessed ASD in children with NF1 visiting a specialized NF1 outpatient clinic without a presumption of ASD. The secondary aim was to investigate the predictive value of a screening instrument and an observational assessment in relation to clinical DSM-IV ASD diagnosis in a pediatric NF1 population. Also, the association of gender, age and intellectual functioning with ASD diagnosis was examined.

## Method

### Participants

Eligible for participation were children (aged 2–10) with either genetically or clinically diagnosed NF1. All children were patients of ENCORE, a multidisciplinary expertise center for genetic neurocognitive disorders (including NF1) in Rotterdam. As part of the standard multidisciplinary care for and follow-up of children with NF1, these children were routinely referred to the Department of Child and Adolescent Psychiatry/Psychology, between August 2011 and August 2016. In the current study, a total of 128 children between 2 and 10 years of age with NF1 were enrolled (45.3% female, mean age = 5.27, *SD* = 1.81).

### Procedure

As standard procedure, all children underwent neuropsychological evaluation and clinical assessment of autistic symptomatology. Additionally, parents and teachers provided information concerning the child’s development and the primary caregiver was asked to complete several questionnaires, including the SRS. The data in this prospective study was collected based on a fixed protocol in the context of the longitudinal follow-up for the assessment of clinical symptoms in children with NF1.

### Measures

#### ASD Symptom Screening

ASD symptoms were screened with the social responsiveness scale (SRS) (Constantino et al. 2003). Completion of the 65 items by one of the parents provides information concerning functioning in the domains social awareness, social cognition, reciprocal social communication, social motivation, and autistic mannerisms. The total raw score, the sum of the 65 items, can be converted into a T-score (*M* = 50; *SD* = 10) using a Dutch normative reference group (Constantino and Gruber 2015). T-scores of 60 or higher indicate mild to moderate problems, and T-scores of 76 or more indicate severe (clinical) problems. The SRS has been shown to be a valid and reliable instrument the scores are independent from IQ scores (Constantino et al. 2003).

#### Observational Assessment of ASD

Observational assessment of ASD was carried out with the autism diagnostic observation schedule-generic (ADOS-G) (Lord et al. 1999) and the autism diagnostic observation schedule—second edition (ADOS-2)(Lord et al. 2012). In most cases (i.e. 88.3%), the ADOS-2 was used. With the ADOS, social interaction, play and imaginative skills are assessed. The ADOS was performed by trained and certified psychologists. Depending on the developmental age and level of expressive language of the child, one of the four available modules of the ADOS was chosen. The ADOS has been shown to be a reliable and valid measure for ASD symptoms (Gotham et al. 2007).

ADOS-G scores were converted to ADOS-2 scores according to the manual (Lord et al. 2012). ASD classifications were obtained and to enable comparison between ASD severity across the four different modules, continuous calibrated severity scores (CSS) were calculated (Gotham et al. 2009; Hus et al. 2014; Hus and Lord 2014). The CSS range from zero to ten with zero indicating no or very little symptoms and ten indicating severe ASD symptoms.

#### Clinical (DSM-IV) Diagnosis

A clinical DSM-IV diagnosis of ASD was established by a multidisciplinary team consisting of a child and adolescent psychiatrist and psychologists, combining information from all assessments, questionnaires, observation of the child and heteroanamnestic information provided by parents and teachers.

#### Intellectual Functioning

Depending on the child’s age, the level of intellectual functioning was assessed with either the wechsler preschool and primary scale of intelligence (WPPSI-III)(Wechsler 2002) or the wechsler intelligence scale for children-iii (WISC-III)(Wechsler 1991). Reliability and validity of these intelligence tests have been demonstrated. Standardized verbal-, performance-, and full scale IQ scores were calculated (*M* = 100, *SD* = 15). For one child, a nonverbal intelligence test (i.e. the Wechsler Non Verbal scale of Ability; WNV) (Wechsler and Naglieri 2006) was used, for which the total IQ score was calculated as well. In two children, assessment with the Wechsler scales was not possible due to a developmental delay. In these children, assessment of intellectual functioning was done using the Bayley Scales of Infant and Toddler Development third edition (Bayley 2006), and a developmental quotient was calculated (developmental age/chronological age × 100, with *M* = 100, *SD* = 15).

### Statistical Analyses

To study the prevalence of ASD, frequencies of the SRS classifications, ADOS-2 classifications, and clinically derived DSM-IV diagnosis were calculated. Sensitivity, specificity, positive predictive (PPV) and negative predictive values (NPV) were calculated to assess the screening accuracy of the instruments’ classifications. The association of intelligence and age with clinically derived DSM-IV ASD diagnosis was examined with independent *t* tests. Missing full-scale IQ scores were imputed using mean imputation. The association of gender with clinically derived DSM-IV ASD diagnosis was examined with a Chi square test. Data were analyzed using IBM SPSS Statistics version 22. Results were considered statistically significant if the (two-tailed) alpha level was below .05. Sensitivity, specificity, PPV and NPV values were interpreted according to the guidelines presented by Cicchetti (2001).

## Results

### Sample Characteristics

Patient characteristics are summarized in Table 1. Of the total sample of 128 children, 58 were female (45.3%). The mean age at assessment of the sample was 5.27 years (*SD* = 1.81). Intelligence scores were significantly lower than the general population mean of 100 (*SD* = 15; total IQ *t*(122) = − 9.40, *p* < .001, verbal IQ *t*(123) = − 6.77, *p* < .001, performance IQ *t*(124) = − 9.24, *p* < .001). Mutations in NF1 were detected as described before (Van Minkelen et al. 2014). The mutations were familiarly inherited in 17.2% (*N* = 22) of the children and de novo in 41.4% (*N* = 53) of the children. In 41.4% of the cases it was unknown whether the mutation was familial or de novo, caused by the fact that parents were not genetically tested, one of the parents was not genetically tested, or the child was not genetically tested yet. The SRS questionnaire was completed by the primary caregiver in 103 children. The mean SRS total T-score was 54.7 (*SD* = 12.60), which is significantly higher compared to the general population mean of 50 (*SD* = 10), *t*(102) = 3.79, *p* < .001. There were no significant differences between the group of children with and without available SRS scores regarding gender, intelligence scores, age, ADOS CSS or DSM-IV diagnosis.

**Table 1:** Patient characteristics

	*N* (%)	Mean (*SD*)	Min	Max
Age at assessment (years)	128	5.27 (1.81)	2	10
Female gender	58 (45.3%)			
Mutation				
Familial	22 (17.2%)			
De novo	53 (41.4%)			
Unknown	53 (41.4%)			
ADOS modules				
Module 1	12 (9.4%)			
Module 2	45 (35.2%)			
Module 3	71 (55.5%)			
ADOS calibrated severity score				
Total	128	2.34 (1.83)	1	8
Social affect	128	2.70 (2.12)	1	9
Restricted/repetitive behaviors	128	3.34 (2.50)	1	10
Type of intelligence test				
WPPSI	98 (76.5%)			
WISC-III	27 (21.1%)			
WNV-III	1 (.8%)			
BSID-III	2 (1.6%)			
IQ scores				
Full-scale IQ	123	88.20 (13.93)	55	119
Verbal IQ	124	91.64 (13.75)	57	124
Performance IQ	125	88.25 (14.21)	58	130
SRS				
Total T-score	103	54.70 (12.59)	35	103

### Autism Prevalence

Of the 128 children with NF1 included in the analyses, 14 received a clinical DSM-IV ASD diagnosis, resulting in a prevalence rate of 10.9%. Based on the SRS, 8.7% of the patients achieved scores in the ‘severe’ (clinical) category (T ≥ 76) and 16.5% in the ‘mild to moderate’ category (T ≥ 60), leading to a total prevalence rate of 25.2%. For the ADOS a total percentage of 18.8% was found, with 10.2% of the children having an autism classification and 8.6% of the children met criteria for an ASD classification. These percentages are lower than the percentages reported earlier with in-depth assessments (Garg et al. 2013; Plasschaert et al. 2015).

### Screening Accuracy

Sensitivity, specificity, positive predictive (PPV) and negative predictive values (NPV) were computed to assess the screening accuracy of the ADOS’ and SRS’ classifications in relation to a DSM-IV ASD diagnosis. The results are displayed in Table 2.

**Table 2:** Sensitivity, specificity, positive predictive value (PPV) and negative predictive value (NPV) of the SRS parental report and ADOS classification compared to DSM-IV classification

	Sensitivity	Specificity	PPV	NPV
SRS class. T ≥ 60	.72	.82	.35	.96
SRS class. T ≥ 76	.46	.97	.63	.95
ADOS class	.64	.89	.45	.95
ADOS class. + SRS class. T ≥ 60	.46	.98	.71	.93
ADOS class. + SRS class. T ≥ 76	.27	.99	.75	.91

With an SRS total T-score cutoff of T ≥ 60 (subclinical), the sensitivity in relation to the DSM-IV diagnosis was fair (.72) and the specificity was good (.82). With a cutoff of T ≥ 76 (clinical), sensitivity was poor (.46) and specificity was excellent (.97). In this population of children with NF1, the PPV was poor (.35) when using the SRS cutoff of T ≥ 60, demonstrating a low probability that a child with NF1 with a positive (T ≥ 60) SRS score is being identified as having ASD according to the DSM-IV. Using this same cutoff of T ≥ 60, the NPV was excellent (.96), indicating a high probability that a child with NF1 with a negative score (T < 60) on the SRS is correctly identified as not having ASD according to the DSM-IV. Using the more stringent (clinical) cutoff of T ≥ 76, the PPV was poor (.63) and the NPV was excellent (.95).

For the ADOS classification, the sensitivity (.64) was poor and the specificity was good (.89). In this population of children with NF1, the PPV (.45) was poor, demonstrating a low probability that a child with NF1 with an ADOS classification is being identified as having ASD according to the DSM-IV. The NPV was excellent (.95), indicating a high probability that a child with NF1 without an ADOS classification is correctly identified as not having ASD according to the DSM-IV.

The sensitivity of the combined classification (classified with ASD on both the ADOS and SRS) was poor using both the T ≥ 60 and T ≥ 76 SRS T-score cutoffs (.46 and .27, respectively). The specificity for both combined classifications was excellent (.98 and .99, respectively). In this population of children with NF1, for both combined classifications the PPV was fair (.71 and .75, respectively) and the NPV was excellent (.93 and .91, respectively).

**Fig. 1:**
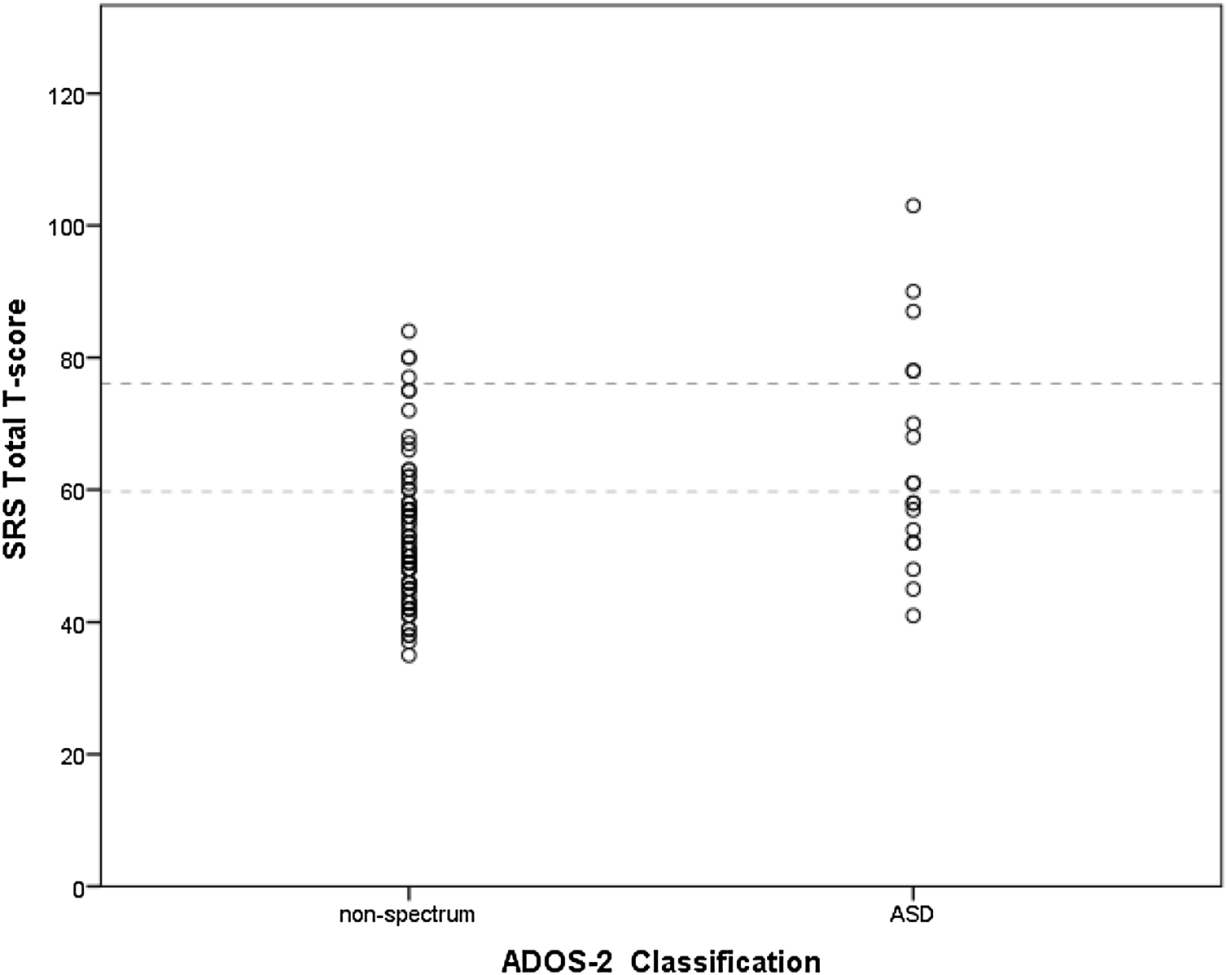
Scatterplot illustrating the distribution of SRS total T-scores (dotted lines indicate the SRS total cutoff scores T ≥ 60 and T ≥ 76) for the two ADOS categories (non-spectrum and ASD), demonstrating low agreement between the instruments

In order to explain the finding of the substantially increased PPV when using the combined classification, an in-depth examination of the distribution of ADOS and SRS scores was performed. The number of participants with an SRS classification (for both T ≥ 60 and T ≥ 76) and ADOS classification were compared to the number of participants with a classification on both instruments. The results are displayed in Table 3. As can be seen, the percentage of classification agreement is low for both the ADOS and SRS T ≥ 60 and the ADOS and SRS T ≥ 76 (i.e. 8.7% and 4.9%). Figure 1 demonstrates that the SRS and ADOS both classify and fail to classify unique cases: a number of children with a SRS score of T ≥ 60 or T ≥ 76 are classified by the ADOS as ‘non-spectrum’ and a number of children with an ADOS ASD classification had a SRS T-score below 60.

**Table 3:** Percentage agreement in ASD classifications between ADOS and SRS

	ADOS	
	Yes	No
SRS T ≥ 60		
Yes	8.7% (9/103)	16.5% (17/103)
No	8.7% (9/103)	66.0% (68/103)
SRS T ≥ 76		
Yes	4.9% (5/103)	3.9% (4/103)
No	12.6% (13/103)	78.6% (81/103)

### Correlates

There were no significant differences in full-scale, verbal, and performance intelligence scores between the group of children with and without DSM-IV ASD diagnosis (Table 2). Significantly more boys (*N* = 11) were clinically diagnosed with ASD according to the DSM-IV criteria compared to girls (*N* = 3). The group of children with a clinical ASD diagnosis was significantly older (mean age = 6.36) compared to the group without diagnosis (mean age = 5.13) (Table 4).

**Table 4:** Correlates DSM-IV diagnosis

	*t*	df	*p*	χ^2^	*p*
Gender	–	–	–	4.05	.044
Full-scale IQ	1.09	111	.28	–	–
Verbal IQ	.62	112	.53	–	–
Performance IQ	1.33	113	.19	–	–
Age	− 2.36	116	.02	–	–

## Discussion

The present study aimed to examine the prevalence of ASD in a sample of children with NF1 without a presumption of autistic symptoms. In our cohort of children with NF1, we found a DSM-IV based ASD prevalence rate of 10.9%. Secondly, we aimed to assess the predictive value of a screening-based and observational instrument on the clinical DSM-IV ASD diagnosis. In this population of children with NF1, combining the classifications from both instruments yielded the highest predictive value on DSM-IV diagnosis. Thirdly, we examined several possible correlates and found a significant effect of gender and age on DSM-IV ASD diagnosis. Our reported DSM-IV based ASD prevalence rate of 10.9% endorses previous reports of an increased prevalence rate in children with NF1 as compared to the general population prevalence of about 0.8% (Baxter et al. 2015), and confirms the involvement of the various mutations leading to NF1 in the development of ASD (Morris et al. 2016). To our knowledge, this is the first study in which ASD prevalence is assessed in a sample of children with NF1 without an initial presumption of ASD. The prevalence reported in this study is likely more representative and accurate for the general pediatric NF1 population than reported by previous studies (Garg et al. 2013; Plasschaert et al. 2015).

Earlier reports of ASD prevalence rates have reported screening-based prevalence rates from 13 to 33% (Adviento et al. 2014; Constantino et al. 2015; Garg et al. 2013; Van Eeghen et al. 2013; Walsh et al. 2013) and prevalence rates based on a observational instrument of 25–26% (Garg et al. 2013; Plasschaert et al. 2015). The results from the observational- and screening instrument demonstrate that not all children with a classification on one of the instruments receive an eventual DSM-IV diagnosis. This implies that a group of children is present with subclinical ASD symptoms in the sample studied here. The percentage of children with screening-based subclinical ASD symptoms (i.e. 16.5%) found in this study supports this. The question for future research remains whether this group of children with NF1 with subclinical ASD symptoms will receive a DSM-IV ASD diagnosis at a later age.

The substantial increase in PPV for the combination of the instruments’ classification scores demonstrates the complementing effect of the two instruments in predicting DSM-IV ASD diagnosis. This might be explained by the difference in the assessment methods (e.g. informant and situation) of the instruments. In case of the SRS, one of the primary caregivers of the child acts as informant for ASD symptoms, whereas in case of the ADOS a trained and certified clinician observes and scores the behavior of the child. Different informants provide valuable and unique information with regard to ASD symptomatology, due to discrepancies in perspective and context, as demonstrated by Duvekot et al. (2015). Moreover, the ADOS assessment is performed in a semi-structured observational lab-setting, whereas the SRS assesses the child’s behavior in daily life. The instruments provide unique information concerning autistic symptoms in the child; the classification agreement between the instruments is low (Table 3) and both instruments classify and fail to classify unique cases (Fig. 1). This underlines the importance of combining the findings from both instruments for ASD assessment in clinical practice.

The group of children with a DSM-IV ASD diagnosis was significantly older compared to the group without diagnosis. It has been demonstrated that children with ASD and other developmental, psychiatric, or neurologic comorbidities are usually diagnosed with ASD at a later age, possibly because the ASD symptoms are ‘masked’ by the comorbidities (Levy et al. 2010) or because assessment and treatment of somatic complaints are prioritized. As a consequence, our sample of children with NF1 might receive a DSM-IV ASD diagnosis at a later age or receive subclinical scores.

Intelligence scores were not related to the DSM-IV ASD diagnosis. This could be due to the lack of dispersion in intelligence scores in our sample, resulting in a homogenous sample regarding cognitive functioning. Examination of gender effects demonstrated an increased number of boys with a DSM-IV ASD diagnosis. This result is in line with the male predominance of ASD in the general population and the presumed female protective effect (Halladay et al. 2015) and with reports of higher prevalence rates of ASD in boys with NF1 (Garg et al. 2016).

A limitation in the current study is that SRS was not available in all children in this study. The parental response rate of 80% might have provided skewed results, since the exact reason for not completing the SRS questionnaire is unknown. However, there were no significant differences between the group of children from responders and non-responders regarding gender, intelligence scores, age, DSM-IV ASD diagnosis or ADOS total CSS, ADOS social affect CSS and ADOS restricted/repetitive behaviors CSS, thus it seems unlikely that differences in SRS scores between responders and non-responders would have been present. Secondly, the combination of the ADOS and ADI-R are generally advocated in ASD research, but the ADI-R was not included in the current study. The data in the current study was initially collected for patient care and the implementation of the ADI-R would have been too time-consuming. Instead, a thorough intake and heteroanamnestic interview with the parents was conducted. Thirdly, the sample used in this study included a number of two- and three year old children and ASD is often diagnosed at a later age. However, the instruments used in the current study are valid instruments for the assessment of autism and autism spectrum disorders in young children. Fourthly, since the data was collected from 2011 onwards, the ASD diagnoses were DSM-IV based instead of DSM-V. Finally, although all children within ENCORE with NF1 were referred to the Department of Child and Adolescent Psychiatry/Psychology for neuropsychological evaluation and assessment of autistic symptomatology, a clinical referral bias might still be present in our sample. The problems experienced in children with NF1 are highly variable, and the severities of the difficulties fluctuate across the population (Lehtonen et al. 2013). Parents of children with for example more severe physical difficulties or developmental delays might shift their focus to these more pressing problems and postpone neuropsychological and behavioral assessment. At the same time, parents of children who experience limited to no difficulties might not see the need for neuropsychological or behavioral assessment. Nevertheless, the sample studied here is a key strength of the study; to our knowledge, this is the first study in which an unselected cohort of children with NF1 is referred for assessment of autistic symptomatology, regardless of an ASD presumption. The relatively large sample size of children with NF1 further strengthens our results. All children underwent uniform neuropsychological and ASD assessments, enabling unique comparisons between different instruments for ASD symptoms and comparison with the eventual DSM-IV ASD diagnosis, as well as the examination of potential correlates with the diagnosis.

## Conclusion

A DSM-IV ASD prevalence rate of 10.9% demonstrates that the prevalence of ASD symptoms in children with NF1 is considerably higher compared to the general population, hereby emphasizing the importance of ASD assessment in this population. Our results underline the relevance of the use of multiple instruments (screening- and observational) for clinicians in order to correctly identify as many individuals with NF1 with ASD as needed. In addition to the group of children with a diagnosis, a substantial group of children with subclinical ASD symptoms is present as well, as was demonstrated by the screening- and observational instrument. This demonstrates the necessity to structurally follow the development of children with NF1.
